# Community-associated Methicillin-resistant *Staphylococcus aureus* and Healthcare Risk Factors

**DOI:** 10.3201/eid1212.060505

**Published:** 2006-12

**Authors:** R. Monina Klevens, Melissa A. Morrison, Scott K. Fridkin, Arthur Reingold, Susan Petit, Ken Gershman, Susan Ray, Lee H. Harrison, Ruth Lynfield, Ghinwa Dumyati, John M. Townes, Allen S. Craig, Gregory Fosheim, Linda K. McDougal, Fred C. Tenover, for the Active Bacterial Core Surveillance of the Emerging Infections Program Network

**Affiliations:** * Centers for Disease Control and Prevention, Atlanta, Georgia, USA;; † University of California, Berkeley, California, USA;; ‡ Connecticut Department of Health, Hartford, Connecticut, USA;; § Colorado Emerging Infections Program, Denver, Colorado, USA;; ¶ Grady Memorial Hospital, Atlanta, Georgia, USA;; # Johns Hopkins Bloomberg School of Public Health, Baltimore, Maryland, USA;; ** Minnesota Department of Health, Minneapolis, Minnesota, USA;; †† University of Rochester, Rochester, New York, USA;; ‡‡ Oregon Health Science University, Portland, Oregon, USA;; §§ Tennessee Department of Health, Nashville, Tennessee, USA

**Keywords:** MRSA, surveillance, antimicrobial resistance, dispatch

## Abstract

To determine frequency of methicillin-resistant *Staphylococcus aureus* infections caused by strains typically associated with community-acquired infections (USA300) among persons with healthcare-related risk factors (HRFs), we evaluated surveillance data. Of patients with HRFs, 18%–28% had a "community-associated" strain, primarily USA300; of patients without HRFs, 26% had a "healthcare-associated" strain, typically USA100.

In the United States, initial reports of methicillin-resistant Staphylococcus aureus (MRSA) infections among injection drug users in Detroit in 1981 were followed by reports of MRSA associated with the deaths of 4 children in Minnesota and North Dakota in 1997 (*1*). For the next few years, public health personnel in several states investigated outbreaks of MRSA infections of skin and soft tissue among diverse populations who typically had little or no previous contact with the healthcare system, such as Native Americans (*2*), sports teams (*3*), prison inmates (*4*), and child-care facility attendees (*5*). These outbreaks were initially associated with a novel MRSA strain known as MW2, or pulsed-field gel electrophoresis (PFGE) type USA400, but were soon replaced by a strain of MRSA belonging to PFGE type USA300 (*6*). Through 2002, the clinical appearance of cases and the microbiologic characteristics of USA300 and USA400 differed substantially from those associated with strains of MRSA acquired in healthcare settings (*7*). Increasingly, MRSA strains of community origin are causing healthcare-associated disease (*8**,**9*). We evaluated surveillance data from a multisite project to determine the frequency with which infections among patients with healthcare-related risk factors (HRFs) were caused by USA300 or other strains of community origin.

## The Study

Active, population-based surveillance for invasive MRSA infections is ongoing in 9 US states (California, Colorado, Connecticut, Georgia, Maryland, Minnesota, New York, Oregon, and Tennessee) through the Active Bacterial Core Surveillance system in the Emerging Infections Program at the Centers for Disease Control and Prevention (CDC). Personnel in each state actively collect laboratory reports of positive MRSA cultures from normally sterile sites (e.g., blood; cerebrospinal, joint, or pleural fluid) of residents in their catchment areas to identify cases. In 2005, the estimated combined population under surveillance was 16.3 million, according to data from the US Bureau of the Census. To report a case, personnel must link a laboratory report to the patient's medical record. During record reviews, personnel abstract information about the following HRFs: culture obtained >48 hours after admission; presence of an invasive device (e.g., vascular catheter, G-tube); and history of MRSA infection or colonization, surgery, hospitalization, dialysis, or residence in a long-term care facility in the 12 months preceding the culture. Case-patients may have >1 HRF. For this analysis, we used information from the record review to classify cases into 3 mutually exclusive groups: 1) case-patients with classic healthcare-associated infections (HA) whose culture was obtained >48 hours after admission with or without other HRFs; 2) case-patients with HRFs but with community onset (i.e., whose cultures were obtained <48 hours after admission) (HACO); and 3) case-patients with community-associated (CA) infections without HRFs, according to medical record review.

A subset of isolates from case-patients was collected from laboratories that voluntarily submitted them for microbiologic characterization. Of the isolates received at CDC by October 2005, a sample of 100 was selected for testing as follows. First, isolates were stratified by Emerging Infections Program site; none were available from Maryland. Second, all isolates from tissues other than blood were selected from each Emerging Infections Program site. To ensure 12–13 isolates per site, we selected blood isolates from case-patients classified as CA and obtained the remainder from samples from HA and HACO case-patients. Isolates were tested by PFGE; patterns were analyzed by using BioNumerics (Applied Maths, Austin, TX, USA). Isolates were grouped into PFGE types using Dice coefficients and 80% relatedness (*10*). We considered isolates with PFGE types USA300, 400, or 1000 to be of community origin and those with types USA100, 200, and 500 to be of healthcare origin as previously described (*10*).

Statistical analysis consisted of comparisons of proportions between CA and HA and between CA and HACO cases using χ^2^ pairwise comparisons. Differences in median age were tested by using Wilcoxon rank sum test.

Of 9,147 cases of invasive MRSA infection investigated from January 2004 through February 2006, 2,535 (28%) were HA, 5,353 (59%) were HACO, and 1,259 (14%) were CA. The median age of case-patients with HA and HACO was significantly higher than that of case-patients with CA (Table 1). CA case-patients were 1) more likely to have pneumonia than HACO but not HA case-patients; 2) more likely to have endocarditis than either HA or HACO case-patients; and 3) less likely to die during this hospital stay than were HA or HACO case-patients.

**Table 1:** Selected characteristics among case-patients with invasive MRSA, by healthcare-related risk factors (HRFs), Active Bacterial Core Surveillance, January 2004–February 2006*

Characteristic	With HRFs, no. (%)		Without HRFs, no. (%)
	Healthcare-associated, n = 2,535	Healthcare-associated, community onset, n = 5,353	Community-associated,† n = 1,259
Median age, y	62‡	62‡	46
Pneumonia	413 (16.3)	685 (12.8)‡	190 (15.1)
Endocarditis	72 (2.8)‡	345 (6.4)‡	158 (12.6)
Died	687 (27.1)‡	845 (15.8)‡	131 (10.4)

*MRSA, methicillin-resistant *Staphylococcus aureus*; HRFs, healthcare-related risk factors. †Patients with community-associated infections were those who did not have HRFs; these patients were used as reference. ‡p<0.05 for χ^2^ test for categorical variables; Wilcoxon rank sum for age.

Of the 100 isolates selected for initial testing, 29 were from HA case-patients, 44 were from HACO case-patients (including 1 isolate of a unique PFGE type), and 27 were from CA case-patients (including 1 isolate that could not be typed) (Table 2). Of the HA isolates, 8 (28%) were USA300. Of the HACO isolates, 6 (14%) were USA300, 1 (2%) was USA400, and 1 (2%) was USA1000. Thus, 18%–28% of isolates in patients with HRFs (HA and HACO) had PFGE patterns typical of community strains. Of the 27 isolates from CA case-patients, 5 (19%) were USA100 and 2 (7%) were USA500; thus, 7 (26%) of isolates among CA case-patients were strains typically considered to be of healthcare origin.

**Table 2:** MRSA isolates from invasive sites by healthcare-related risk factors (HRFs) and PFGE type, Active Bacterial Core Surveillance, January 2004–February 2006*

PFGE type	Healthcare associated, no. (%)	Healthcare associated, community onset, no. (%)	Community associated,† no. (%)	Total, no. (%)
USA100	20 (69)	30 (68)	5 (19)	55 (55)
USA200	1 (3)	0	0	1 (1)
USA300	8 (28)	6 (14)	18 (67)	32 (32)
USA400	0	1 (2)	0	1 (1)
USA500	0	5 (11)	2 (7)	7 (7)
USA1000	0	1 (2)	1 (4)	2 (2)
Unique type	0	1 (2)	0	1 (1)
Not typeable	0	0	1 (4)	1 (1)
Total	29 (100)	44 (100)	27 (100)	100 (100)

*MRSA, methicillin-resistant *Staphylococcus aureus*; PFGE, pulsed-field gel electrophoresis. †Patients with community-associated infections were those who did not have HRFs.

## Conclusions

MRSA strains such as USA300, which were initially a cause of MRSA infections in the community, have migrated into healthcare settings. The results from this multisite project are consistent with observations from individual facilities, where USA300 isolates caused illness in patients whose infection was healthcare associated (*11**,**12*). Although age and frequency of endocarditis still differed between case-patients with HRFs (HA and HACO) and those without HRFs (CA), PFGE testing indicated that 18%–28% of patients with HRFs were infected with a "community-associated" strain of MRSA, primarily USA300. Furthermore, 26% of patients without HRFs had a "healthcare-associated" strain, typically USA100. Thus, the distinction between healthcare- and community-associated MRSA is rapidly blurring.
